# Sense of meaning in life, self-acceptance, and prosocial behavior: an application of network analysis methods

**DOI:** 10.3389/fpsyg.2025.1533687

**Published:** 2025-04-07

**Authors:** Li Guo, Yangtong Niu, Xinying Li, Yuting Li, Zhaoxia Xue, Guane Yang

**Affiliations:** ^1^School of Humanities and Social Sciences, Shanxi Medical University, Taiyuan, China; ^2^School of Pharmaceutical Sciences, Shanxi Medical University, Jinzhong, China

**Keywords:** college students, sense of meaning in life, prosocial behavior, self-acceptance, analysis methods

## Abstract

**Objective:**

To explore the characteristics and core items of the network structure between school students’ sense of meaning in life, self-acceptance, and prosocial behavior, and to provide a basis for understanding the relationship between their sense of meaning in life, self-acceptance and prosocial behavior and related interventions.

**Methods:**

A survey of 1232 school students was conducted using the Self-Acceptance Scale, the prosocial Behavior Scale, and the Sense of Meaning of Life Scale. Network analysis was used to construct the network of prosocial behavior, self-acceptance, and sense of the meaning of life among school students, and the software R was used for statistical analysis and visualization.

**Results:**

In the regularized bias correlation network of self-acceptance, prosocial behavior, and sense of meaning in life among school students, self-acceptance and self-appraisal, having meaning and self-appraisal, anonymity and altruism had the strongest correlation; emotionality, altruism, and urgency had the highest expected impact; and having meaning and self-appraisal had the highest expected impact of the bridge.

**Conclusion:**

Self-acceptance, meaning in life, and prosocial behavior are interrelated; interventions targeting emotionality, altruism, and urgency in the prosocial behavior dimensions may maximize prosocial behavioral effects among college students.

## 1 Introduction

Prosocial behavior has a positive effect on individuals to maintain good interpersonal relationships and social reputation, as well as to promote the development of human social undertakings, can enhance social ties, and is conducive to the construction of a more harmonious social environment (Yuan et al., 2016). Under the framework of “human flourishing” proposed by positive psychology (Fredrickson, 2001), prosocial behavior facilitates individual well-being and social capital accumulation through its capacity to strengthen social connectedness and emotional resonance (Grant and Gino, 2010). Against the backdrop of escalating global conflicts, the altruistic motivation inherent in such behaviors and their peace-building potential offer mechanism-level psychological solutions for cultivating cross-cultural understanding (Böckler et al., 2016). Existing research has empirically established robust associations between prosocial behavior and positive psychological constructs including meaning in life (Steger et al., 2008a,b) and self-acceptance (Chang et al., 2024). However, the precise mechanisms through which these psychological resources dynamically interact to co-shape prosocial behavioral patterns remains a critical theoretical gap demanding systematic investigation in contemporary positive psychology scholarship.

Prosocial behavior is kind and caring behavior toward others (Flook et al., 2015), constitutes a core manifestation of positive psychological functioning. It serves not only as a critical determinant in maintaining individual social adaptation and psychological well-being (Seligman and Csikszentmihalyi, 2000), but also functions as a foundational cornerstone propelling the prosocial advancement of human societies. College students’ mental health, social skills, and academic performance are all impacted by prosocial activity (Wentzel, 1993), and it can also help them build positive social interactions with their classmates and family (Cheng et al., 2018). Previous studies have shown that many factors influence prosocial behavior; in addition to the external environmental stimuli (Li et al., 2019) and the individual’s perception of the event (Zhang and Zhang, 2014), it is inextricably linked to the individual’s emotions and personality and other factors (van Kleef and Lelieveld, 2021). The social environment, cognition, and emotion of college students show rapid development and variable phenomena, so the college period is a critical period for the development of individual emotion and sociality (including prosocial behavior) (Goldstein et al., 2015). At this critical stage of development, the prosocial behaviors of college students are particularly noteworthy, and the introduction of network analysis methods can precisely reveal the interaction patterns and propagation paths of these behaviors in complex social networks. It allows us to investigate in greater detail how these habits spread and evolve throughout the social networks of college students (Hevey, 2018).

The sense of life meaning, a core construct in positive psychology, comprises two dimensions—meaning presence and meaning search—corresponding to the static cognition and dynamic process of individual existential experiences, respectively (Steger et al., 2006). Meaning having refers to an individual’s feeling that his or her life has meaning while meaning seeking refers to an individual’s motivation and goal to pursue and explore meaning in the course of life, and both experiences are crucial to an individual’s sense of well-being and fulfillment (Dezutter et al., 2013). Several longitudinal studies have shown that a sense of meaning in life positively predicts life satisfaction as well as higher quality of life and reduces individuals’ severe internalizing problems (e.g., depression) and externalizing problems (e.g., problematic Internet use) (Ye et al., 2021). In addition, a sense of meaning in life can provide positive social benefits and promote prosocial behavior. Research suggests that a sense of purpose in life may inspire individuals and influence their current actions (Yang et al., 2016). Based on the Investigation of Terror Management Theory and Existential Significance, a person’s psychological well-being and social functioning may be preserved by using a sense of purpose as a psychological tool to guard against various actual and potential existential dangers effectively (Reker and Chamberlain, 1999). This aligns with Viktor Frankl’s perspective in “Man’s Search for Meaning, “where he observed that even in the extreme conditions of Nazi concentration camps, individuals were able to overcome fear and uncertainty by finding meaning in life. Frankl emphasized that even under the most adverse circumstances, people retain the capacity to choose their attitude and behavior. This powerful realization can empower us in our own lives, manifesting what he termed “the courage to be (Frankl, 2015). People are more inclined to take prosocial actions to discover their purpose in life. Put another way, when individuals are more motivated to find the meaning of life, they are more likely to regard themselves as givers, help others, and contribute to society (Baumeister et al., 2013). According to self-determination theory (Ryan and Deci, 2000), when individuals fulfill their relatedness needs through prosocial behaviors, their life meaning system receives positive feedback, potentially forming a dynamic cycle that drives positive psychological development (Klein, 2017).

In summary, we can speculate that there is a strong link between the sense of meaning in life and prosocial behavior. However, existing research also reveals that the relationships between prosocial behavior and sense of meaning in life differ (Liu, 2022), suggesting the need for a more in-depth exploration of the interactions between the two.

Self-acceptance refers to an individual’s adaptive attitude toward their own characteristics and social roles, encompassing a dialectical cognition of strengths and weaknesses (Sun and Lu, 2017), which involves the dimensions of self-evaluation and self-acceptance (Cong and Gao, 1999). Self-acceptance enhances positive emotional experiences (Fredrickson, 2001), thereby stimulating individual behavioral motivation. Upon surpassing a critical threshold of positive emotions (Meng et al., 2017), a “broaden-and-build” effect is triggered, prompting individuals to seek positive experiences and attend to others’ needs actively (Li et al., 2019), ultimately translating into specific prosocial behaviors (Li et al., 2024). Self-acceptance and self-compassion share an inclusive attitude toward the self (Yang and Tong, 2023), which reduces self-criticism and enhances self-worth (Chang et al., 2024), thereby improving mental health levels and storing psychological energy for prosocial behaviors (Zhang et al., 2024; Hu, 2020; Popov et al., 2016).

Self-compassion is predictive of prosocial behaviors (Chang et al., 2024; Tao et al., 2023; Ye et al., 2021). Thus, the present study hypothesized that self-acceptance may have the same effect. Additionally, individuals who accept themselves are more likely to establish a sense of social belonging (Baumeister and Leary, 1995), which drives them to actively uphold collective well-being (Han, 2019; Neff and Beretvas, 2013).

As a core dimension of positive self-views (Ryff, 1989), self-acceptance not only mitigates the emotional depletion caused by self-criticism (Neff, 2011) but also enhances psychological resilience, providing energy reserves for sustained altruistic behavior (Zeng et al., 2015). Notably, existing research predominantly adopts a variable-centered approach to examining the predictive role of single psychological resources on prosocial behavior (Martela and Ryan, 2015), neglecting the potential networked synergistic mechanisms among positive psychological elements—a critical theoretical gap this study aims to address.

The current study suggests using network analysis to further explain the connection between prosocial conduct, self-acceptance, and a feeling of purpose in life. Traditional statistical methods, such as structural equation modeling or regression analysis, commonly posit that the relationships between variables are linear and unidirectional, relying on latent variables to account for the covariation among observed variables (Borsboom, 2008). Nevertheless, these methodologies exhibit significant limitations in capturing the dynamic interactions and non-linear relationships characteristic of multidimensional psychological constructs. For instance, the relationship between self-acceptance and prosocial behavior may be indirectly linked through a sense of life meaning, while also potentially involving direct feedback effects—a level of complexity that traditional modeling techniques are ill-suited to represent simultaneously. In contrast, network analysis dispenses with the assumption of latent variables, instead conceptualizing psychological phenomena as dynamic systems comprised of nodes (variables) and edges (relationships), thereby providing a more comprehensive and nuanced framework for understanding such intricate psychological dynamics (Borsboom and Cramer, 2013). Through the application of regularized partial correlation networks, this study was able to identify direct associations between variables and quantify the centrality and bridging effects of nodes within the network (such as “sense of life meaning” serving as a critical bridge connecting self-acceptance with altruistic behavior). This data-driven analytical approach is particularly well-suited for exploring the synergistic interactions among multiple variables that are not fully explained by existing theories (Robinaugh et al., 2019), thereby providing a more congruent analytical framework for the complex psychological phenomena under investigation in this research. The network analysis approach allows researchers to explore the interrelationships among the individual elements of the variables of interest and helps to identify bridging symptoms in the network (Kaiser et al., 2021; Wu et al., 2024). Research suggests that interventions targeting bridging symptoms across networks may be more effective (Jones et al., 2021). Thus, identifying bridging symptoms in prosocial behavioral network models is critical to increasing the likelihood of prosocial behavior among college students (Robinaugh et al., 2019). This approach circumvents traditional *a priori* assumptions about variable relationships by utilizing regularized partial correlation networks (e.g., the EBICglasso model) to extract robust associations (Costantini et al., 2015). It facilitates the exploration of non-linear relationships between self-acceptance, prosocial behavior, and a sense of life meaning that are not fully explained by existing theory (Boccaletti et al., 2006). In summary, we obtained evidence on the interconnections between a sense of meaning in life, self-acceptance, and prosocial behavior. Nonetheless, previous research has not fully explored the associations between these constructs in the same study, nor have they deeply analyzed their unique interactions. Given the complex pattern of interrelationships between self-acceptance, sense of meaning in life, and prosocial behavior, understanding the associations between these constructs, more research is still necessary to fully reveal the relationships between these concepts. In this context, network analysis, an analytical technique used to study patterns of interrelationships and interactions between entities, can map interactions between different data-driven constructs (Bringmann and Eronen, 2018). It provides a unique perspective for studying complex relationships between several factors (Costantini et al., 2015). More importantly, by providing alternative and insightful ways to account for patterns of connectivity between variables of interest and to reflect the centrality of a given variable in the network (e.g., using Fruchtermann and Reingold’s algorithm: Fruchterman and Reingold, 1991), network analysis allows for intuitive understanding of the model’s of complex structures (Bringmann and Eronen, 2018).

The research aim of the present study was to investigate for the first time the complex model of the relationship between self-acceptance, a sense of meaning in life, and prosocial behavior and the relationship between them, using a network analysis approach.

## 2 Data and methods

### 2.1 Subjects

This study was measured from July 10, 2024, to October 9, 2024, and the questionnaires were distributed and retrieved using a convenience sampling method through the Brain Island platform and Questionnaire Star platform. The study was approved by the Ethics Committee of Shanxi Medical University, and subjects were considered to have signed an informed consent form when they began to answer the questions. To ensure that the questionnaire survey results were true and reliable, clear instructions were given to explain the purpose, significance, and precautions of the questionnaire survey to the subjects, and the subjects filled in anonymously to eliminate doubts. Two master examiners verified the recovered questionnaires, and the exclusion criteria were: ① insufficient thinking about the polygraph topic; ② data blanks, errors, and obvious patterns. A total of 1,232 valid questionnaires were recovered.

### 2.2 Methods

#### 2.2.1 Meaningfulness of life scale

American social psychologist Steger et al. (2006) believed that the sense of meaningfulness of life includes two dimensions, namely, the meaning of life perception (MLQ-P) and meaning of life searching (MLQ-S). They compiled a meaning of life scale (meaning of life scale) based on these dimensions. Meaning in life questionnaire (MLQ) was developed on this basis. The MLQ is scored according to the degree of conformity to the description of the question, ranging from 1 to 7 on a scale from “not at all conforming” to “fully conforming.” This scale is used to study the individual’s experience and pursuit of life meaning, and the higher the score, the higher the individual’s meaning. In this study, the a-coefficient of the total scale is 0.861, and the a-coefficients of the two dimensions of the Meaning of Life Perception (MLQ-P) and the Meaning of Life Seeking (MLQ-S) are 0.890 and 0.901, respectively, which meet the psychometric standards.

#### 2.2.2 Self-acceptance questionnaire

The Self-Acceptance Questionnaire (SAQ) was developed by scholars Cong and Gao (1999), and it can be applied to measure and rate the self-acceptance characteristics of subjects. The SAQ consists of two factors, self-evaluation and self-acceptance, with a total of 16 entries (each factor consists of 8 entries), and a 4-point scale ranging from “1 = very opposite” to “4 = very much the same” and a 4-point scale ranging from “1 = very opposite” to “4 = very much the same.” The scale was rated on a 4-point scale from “1 = very opposite” to “4 = very much the same,” with the higher the total score, the better the individual’s self-acceptance. This scale’s reliability and validity test found that the coefficient was 0.888, with a self-acceptance factor of 0.862 and a self-evaluation factor of 0.876.

#### 2.2.3 Prosocial behavior scale

Using the prosocial Tendencies Scale, developed by Gustavo Carlo in 2002 (Zhang et al., 2015), the scale consists of 26 questions ranging from “very unlike me” to “very much the same.” The scale consists of 26 questions, ranging from “very unlike me” to “very much like me,” with scores increasing from 1 to 5. The scale examines the tendency to engage in prosocial behavior in public, in anonymous situations, where others are explicitly or implicitly present, in purely altruistic situations, in emergencies, and when emotions and feelings are aroused. The higher the score for each category, the stronger the tendency to behave prosocially. The total a-coefficient of the scale was 0.928; public situations: 0.868, anonymous situations: 0.891, adherence: 0.849, pure altruism: 0.815, emergencies: 0.680, and emotional and affective arousal: 0.824, and the total score of the scale and its dimensions had good reliability.

### 2.3 Data were analyzed using R software

Data were analyzed using R software (version 4.4.1, open source, available at https://www.r-project.org/). The association between prosocial behavior, sense of meaning in life, and self-acceptance among college students was explored. The study utilized (1) regularized partial correlation networks to identify indicators of node centrality and predictability, which can help identify key intervention targets (Robinaugh et al., 2016). Analyses were designed to identify undirected network consistency and variability with directed networks to guide early intervention goals. Descriptive statistics were calculated in SPSS, and count data were reported as number of cases, mean, and standard deviation. For the first analysis, a Gaussian graphical model (GGM) was used for data fitting and item network construction (Epskamp et al., 2018). The GGM is an undirected network, with the nodes representing the observed variables (2 dimensions from the Sense of Meaning in Life Scale, 6 dimensions of prosocial behavior, and 2dimensions of self-acceptance in the present study) and the connecting line between two nodes representing their partial correlations (Epskamp and Fried, 2018). The data were non-supernaturally transformed using a huge software package (Tuo et al., 2012) to account for the multivariate normal distribution assumption of the GGM. The least absolute shrinkage and selection operator (LASSO) and the extended Bayesian information criterion (EBIC) were used to refine the network edges and tune the parameters to enhance the interpretability (Epskamp et al., 2018) by normalizing the network by shrinking the very small associations to zero. The nodes represent the variables, while the edges’ thickness and color represent the association’s degree and potency, respectively (Zhang S. et al., 2022; Zhang X. et al., 2022). In visualization networks, green edges indicate a positive correlation and red edges indicate a negative correlation. Thicker edges indicate a stronger correlation between nodes. A circle around the outside of anode indicates the predictability of that node, and the closer to completing the filling represents the higher explanation rate of the node to the neighboring nodes (Wu et al., 2022). Following the suggestion of Epskamp et al. (2017), we evaluated the edge accuracy using the bootstrapped method. The above network construction and visualization were implemented via the graph package and bootnet.

Next, to quantify the importance of each node in the network, we calculated the centrality index (Opsahl et al., 2010). Centrality indices indicate the degree to which a node is connected to the rest of the network and may indicate influential initial treatment goals. Centrality indices – strength and expected impact – were calculated to determine the importance of each variable (Epskamp et al., 2018).

Strength was calculated by summing the absolute value of the weights of all edges connected to a node. To determine confidence intervals (CIs) for each edge’s strength and the centrality metric’s stability, we computed 10,000 bootstrapped networks. The bridge expected impact of each node, and thus the bridge symptom, was computed using the MGM package, with the bridge expected impact defined as the sum of the values of all edges connecting a given node to nodes in other symptom clusters. Higher values of bridge expected impact indicate a greater degree of increased risk of transmission to other symptom clusters (Jones et al., 2021). Centrality is a relative measure of node influence, whereas predictability is absolute (Epskamp et al., 2018). That is, predictability gives an absolute quantification of the variance accounted for by all other nodes in the network for a given node. At the same time, intensity quantifies interconnectivity relative to other nodes in the network.

Referring to Constantin’s suggestion (Constantin et al., 2023), when the network consists of 20 or fewer nodes, a sample size of 250 ~ 350 is usually sufficient to observe moderate sensitivity, high specificity, and high side-weight correlation. We distributed 1,305 questionnaires, and after screening and excluding invalid ones, we ultimately analyzed 1,232 valid questionnaires, achieving a response rate of 94.41%.

## 3 Results

### 3.1 Common method bias test

The Harman one-way test for common method bias was used, and the results showed nine factors with eigenvalues greater than 1. The explanation rate of the first factor was 23.80%, which is lower than the critical value of 40%, indicating no serious common method bias in this study (Aguirre-Urreta and Hu, 2019).

### 3.2 Descriptive statistics

#### 3.2.1 Descriptive statistics among variables

Table 1 contains the mean and range of variables. A total of 1,232 participants (762 females and 470 males) were included in this study, and the mean age of the participants was (24.36 s 4.81) years.

**Table 1:** Descriptive statistics of variables (*N* = 1,232).

Variant	Averages	Standard deviation	Minimum value	Maximum values
Self-acceptance	23.01	5.49	8	32
Self-esteem	20.48	4.57	8	32
Have a sense of meaning	23.50	6.49	5	35
Seek a Sense of Meaning	26.28	5.35	5	35
Openness	13.70	3.20	4	20
Anonymity	18.66	4.00	5	25
Altruism	15.52	2.78	4	20
Compliance	18.64	3.35	8	25
Emotional	18.84	3.25	7	25
Urgency	12.16	1.80	5	15

#### 3.2.2 The test of differences in gender for each variable

To explore the differences in gender for self-acceptance, prosocial behavior, and sense of meaning in life, an independent samples *t*-test was used for the analysis, and the results showed that boys scored significantly higher than girls in terms of the total score of self-acceptance, prosocial behavior, and sense of meaning in life, as well as in terms of their dimensions (see Table 2).

**Table 2:** Analysis of differences in variables by gender (*N* = 1,232).

Variant	Male	Female	*t*	Cohen’s *d*
Self-acceptance	23.77 ± 5.56	22.54 ± 5.40	3.81^***^	0.11
Self-esteem	21.45 ± 4.79	19.88 ± 4.34	5.90^***^	0.17
Have a sense of meaning	24.98 ± 6.52	22.58 ± 6.31	6.40^***^	0.18
Seek a sense of meaning	27.00 ± 5.59	25.83 ± 5.16	3.74^***^	0.11
Openness	14.28 ± 3.32	13.34 ± 3.06	5.03^***^	0.14
Anonymity	19.17 ± 4.31	18.36 ± 3.77	3.53^***^	0.10
Altruism	15.73 ± 3.08	15.40 ± 2.57	2.04^***^	0.06
Compliance	19.31 ± 3.48	18.23 ± 3.19	5.61^***^	0.16
Emotional	19.31 ± 3.41	18.56 ± 3.12	3.95^***^	0.11
Urgency	12.33 ± 1.95	12.05 ± 1.68	2.62^***^	0.07
Total self-acceptance score	45.21 ± 8.70	42.43 ± 8.16	5.68^***^	0.16
Sense of the meaning of life	51.98 ± 10.21	48.42 ± 8.88	6.46^***^	0.18
Prosocial behavior	100.12 ± 15.37	95.92 ± 12.76	5.19^***^	0.15

*p < 0.5, **p < 0.1, and ***p < 0.001.

### 3.3 Regularized biased correlation network

**Figure 1:**
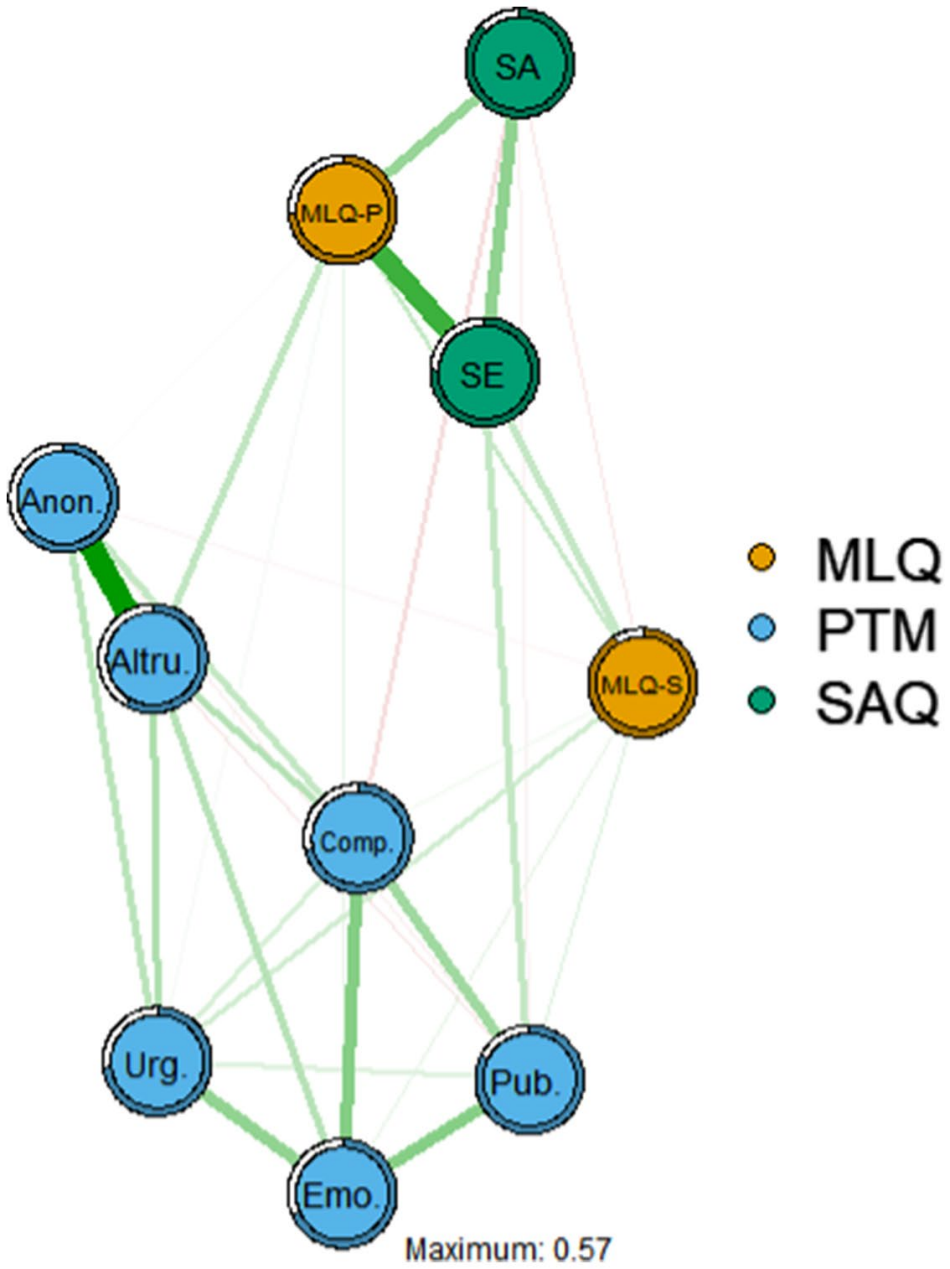
Regularized partial correlation network. Maximum Absolute Edge Strength = 0.57. The thickness of the edges indicates the strength of the association between constructs. Green/full edges indicate positive associations and red/dashed edges indicate negative associations. SA, self-acceptance; SE, self-evaluation; MLQ-P, having a sense of meaning; MLQ-S, seeking a sense of meaning; Pub., publicity; Anon., anonymity; Altru., altruism; Comp., compliance; Emo., emotionality; Urg., urgency. The regularized bias correlation values are shown in the results section of the text below. The edge weights given in the model are shown in Table 3.

The regularized biased correlation network is shown in Figure 1. The network’s strongest edges are self-acceptance and self-evaluation, meaning and self-evaluation, and anonymity and altruism. Self-evaluation was most strongly correlated with having meaning, and having meaning was significantly correlated with altruistic behavior.

Self-evaluation was positively correlated with publicity, while self-acceptance was conditionally independent of publicity. In addition, having meaning was positively correlated with self-acceptance and self-evaluation, and having meaning was positively correlated with altruism and adherence to prosocial behavior.

Self-evaluation was conditionally independent of altruism and anonymity in prosocial behavior after considering all associations in the network.

Altruism positively correlated with meaning in anonymity and a sense of meaning in life.

The correlation and edge weight matrices are reported in Table 3 (for edge accuracy, which contains confidence intervals for each edge shown in the model).

**Table 3:** Correlation and edge weight matrix (*N* = 1,232).

	1	2	3	4	5	6	7	8	9	10
1	–	0.415^**^	0.406^**^	0.064^*^	0.03	0.055	0.087^**^	−0.013	0.081^**^	0.058^*^
2	0.24	–	0.597^**^	0.290^**^	0.262^**^	0.184^**^	0.239^**^	0.191^**^	0.246^**^	0.291^**^
3	0.23	0.43	–	0.299^**^	0.200^**^	0.291^**^	0.367^**^	0.244^**^	0.313^**^	0.291^**^
4	−0.05	0.13	0.12	–	0.243^**^	0.151^**^	0.202^**^	0.237^**^	0.275^**^	0.275^**^
5	−0.03	0.13	–	0.07	–	0.202^**^	0.243^**^	0.443^**^	0.493^**^	0.355^**^
6	–	–	0.01	−0.02	−0.04	–	0.758^**^	0.514^**^	0.466^**^	0.537^**^
7	–	–	0.13	–	−0.06	0.57	–	0.561^**^	0.550^**^	0.582^**^
8	−0.07	–	–	0.03	0.21	0.13	0.16	–	0.600^**^	0.505^**^
9	–	0	0.05	0.05	0.27	–	0.16	0.27	–	0.571^**^
10	–	–	0.02	0.1	0.07	0.15	0.17	0.09	0.24	–

The upper right half of the table shows the correlation matrix and the lower left half of the table shows the GGM weight matrix. 1 = Self-acceptance, 2 = Self-appraisal, 3 = Possessing meaning, 4 = Seeking meaning, 5 = Openness, 6 = Anonymity, 7 = Altruism, 8 = Dependence, 9 = Emotionality, 10 = Urgency, *p < 0.5, **p < 0.1.

**Figure 2:**
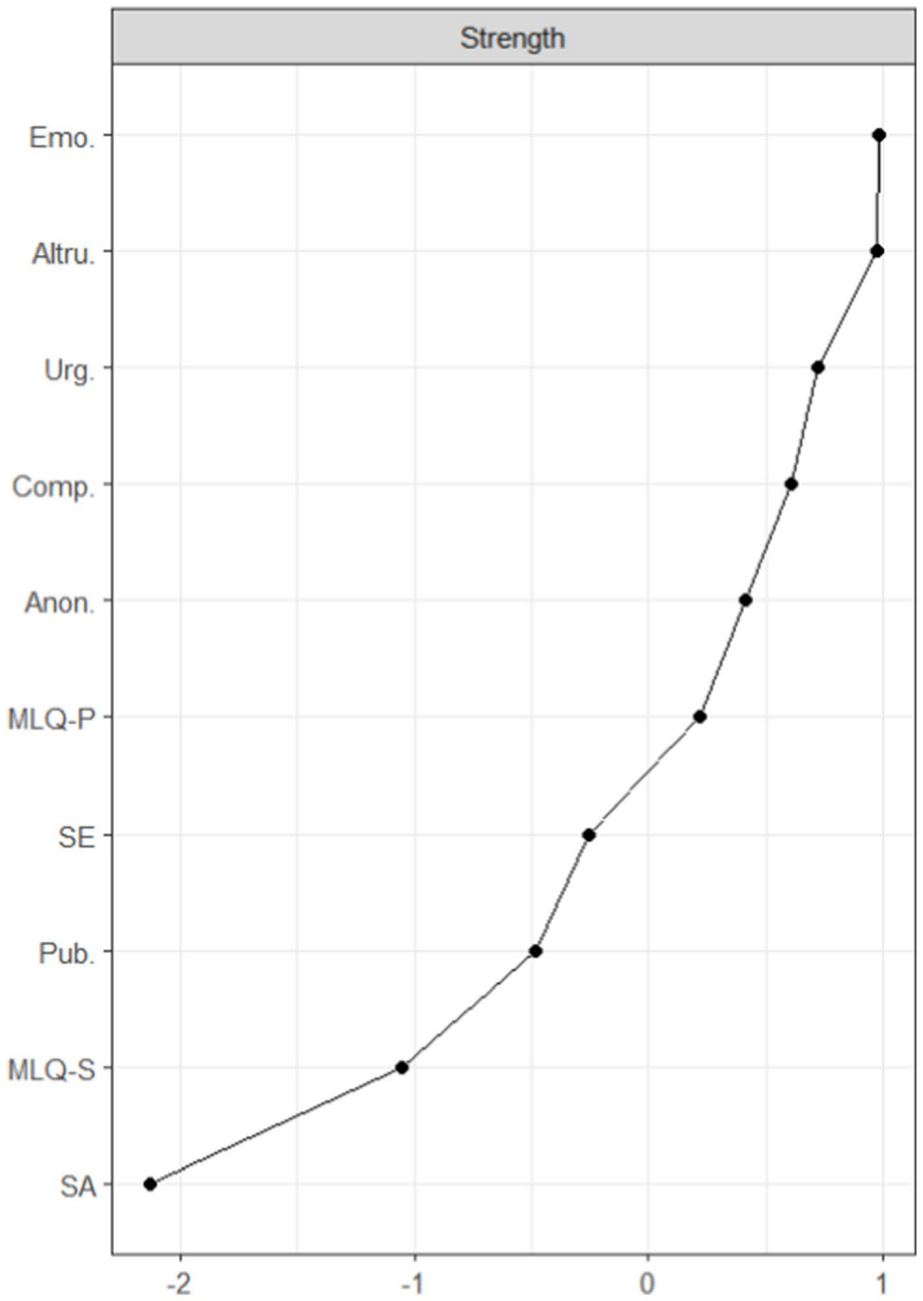
Standardized centrality index. This figure ranks the nodes included in the network model according to the node strength (connection strength) level, indicating the extent to which these nodes occupy a more central position in the network. Emotionality and altruism are the most central nodes in the model.

Stability analyses of the mean connection strengths for the prosocial behavior dimensions of emotionality and altruism showed good stability for intensity centrality (correlation stability coefficient = 0.57). This suggests that the order of nodes in the intensity centrality dimension remained similar when a significant portion of the sample was discarded (Figure 2).

**Figure 3:**
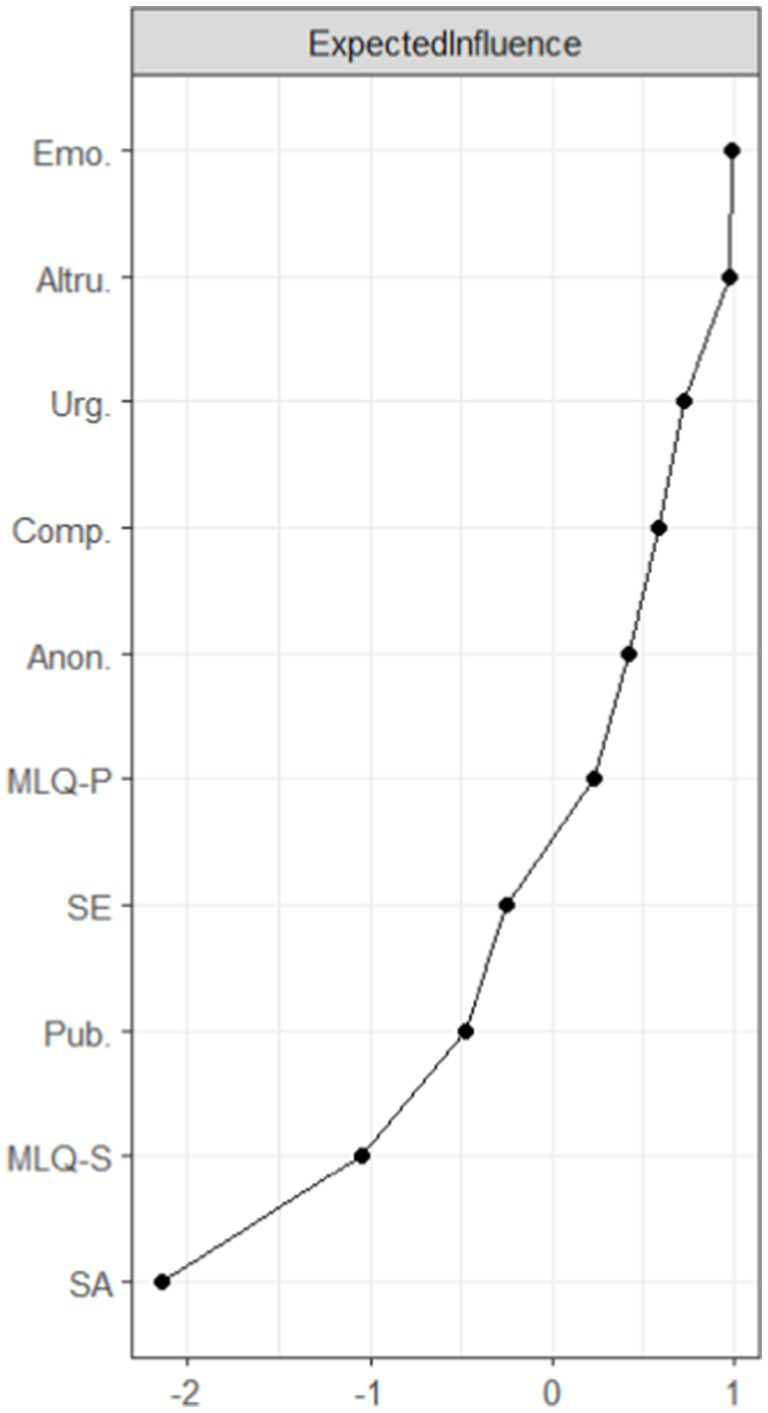
Map of expected impact results.

The magnitude of the expected impact of the nodes is shown in Figure 3. The horizontal axis represents the magnitude of the expected impact value, with the closer to the right side indicating a higher expected impact. Emotionality, altruism, and urgency have the highest expected impacts at 3.60, 3.59, and 3.39, respectively, indicating that they are statistically the most widely associated symptoms in the structure of this network. The correlation stability coefficient for the expected impact of nodes is 0.750, indicating that the estimates of the expected impact of nodes are sufficiently stable.

**Figure 4:**
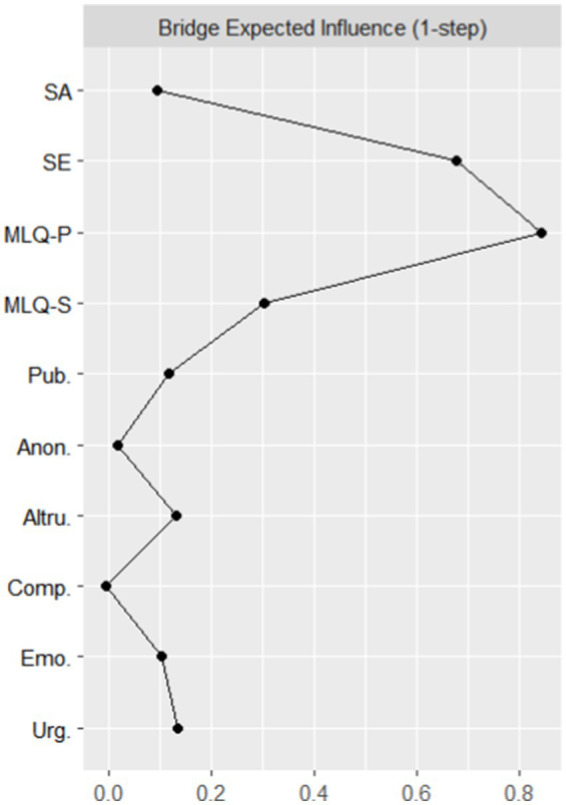
Map of expected bridge impact results.

The magnitude of the bridge’s expected influence on the nodes is shown in Figure 4, where the axes represent the magnitude of the bridge’s expected influence values, and the closer to the right side, the higher the bridge’s expected influence. Possessing meaning and self-evaluation have the highest bridge expected impacts of 0.84 and 0.68, respectively, which suggests that in the existing network structure, prosocial behaviors can be maximally influenced by “possessing meaning,” and “self-evaluation” can maximally improve the sense of life meaning. Maximize improving the sense of meaning in life through “self-evaluation.” The stability coefficient associated with the expected impact of node bridges is 0.75, indicating that the estimates of the expected impact of node bridges are relatively stable.

The node predictability metric is visualized as a ring around a node in Figure 1, with a larger percentage of the colored portion of the ring indicating greater predictability of the item. The results in Table 4 show that the average of all node predictability values from 0.17–0.66 is 0.43. This means that, on average, 43.57% of the current node variance in the network can be explained by its neighboring nodes. In the model, the bridge of meaning presence is anticipated to have the highest impact value, followed by self-evaluation.

**Table 4:** Centrality indicators.

	Predictability	Expected impact (in *Z*-scores)	Anticipated impact of the bridge
SA	0.23	−2.15	0.1
SE	0.43	−0.25	0.68
MLQ-P	0.46	0.23	0.84
MLQ-S	0.17	−1.04	0.3
Pub.	0.33	−0.47	0.12
Anon.	0.60	0.42	0.02
Altru.	0.66	0.98	0.13
Comp.	0.49	0.58	0
Emo.	0.53	0.99	0.1
Urg.	0.47	0.72	0.13

## 4 Discussion

### 4.1 Analysis of differences in self-acceptance, sense of meaning in life, and prosocial behavior

The results of the present study showed that boys scored significantly higher than girls in the total scores of self-acceptance, sense of meaning in life, and prosocial behavior as well as their scores on each of the dimensions, which is consistent with previous studies (Zhang, 2023; Zhang, 2017; Barchia and Bussey, 2011). To gain a profound understanding of this phenomenon, a meticulous discussion will be conducted, delving into the intricate interplay of cultural and social contexts.

In the traditional Chinese educational system, a suppression-oriented cultivation model is prevalent for male children. This model, which involves comparison with others and emphasizes gender role stereotypes, plays a pivotal role in enhancing males’ psychological resilience to adverse evaluations. It shapes a defense mechanism characterized by “low expectations and high psychological resilience.” From an early age, males are instilled with values of strength, endurance, tolerance, and humility. They are culturally expected to suppress emotional expression even in the face of grievance to avoid public ridicule, thereby reinforcing the formation and consolidation of this defense mechanism. The interplay of this educational model and cultural expectations profoundly influences the psychological development trajectory of male individuals (Zhang, 2017). Consequently, males tend to exhibit a slightly higher level of self-acceptance compared to females.

According to gender role theory, society holds distinct role expectations for males and females. Males are typically expected to act as protectors and providers, a societal pressure that prompts male students to engage more in prosocial behaviors. This pressure to align with societal role expectations is a significant influence on male students. Additionally, male students may demonstrate prosocial behaviors to assert their social status and strength. In contrast, female students are more encouraged to exhibit caring and cooperative behaviors, which, although prosocial, are not always viewed as typical prosocial behaviors by traditional perspectives (Chen, 2017). Additionally, the Chinese educational system and parenting styles may differ in the upbringing of male and female students. Greater emphasis is often placed on cultivating a sense of responsibility and public awareness in male students, which may be a significant factor contributing to their higher scores in prosocial behaviors (Song et al., 2019).

According to existential theory, a sense of meaning in life relates to an individual’s self-actualization and goal-seeking. Men may be encouraged to pursue personal achievement and independence during socialization. The attainment of these goals may contribute to a greater sense of meaning. In contrast, women may be encouraged to focus more on interpersonal relationships, resulting in a more diverse source of meaning, but may not necessarily be socially recognized as “meaning-rich” (Fabry, 1980). Emerging research in psychological science suggests gender-based divergences in emotional regulation and stress-coping mechanisms, with practical implications for professionals in the field. Males tend to prioritize problem-focused cognitive appraisals over emotional rumination, a proactive coping strategy associated with enhanced psychological well-being and meaning-making processes. The empirical evidence indicates that male adolescents facing academic setbacks frequently employ analytical problem-solving approaches rather than persistent negative affective states, as demonstrated in longitudinal studies of adaptive functioning (Platt et al., 2023).

### 4.2 Cross-sectional network analysis of self-acceptance, sense of meaning in life, and prosocial behavior

This study, which utilized network analysis, delved into the intricate interplay among self-acceptance, sense of meaning in life, and prosocial behavior in college students. The network analysis revealed a robust network model, indicating a close connection between the variables. These findings are of utmost importance, as they suggest that Emotionality, Altruism, and Urgency are central hubs in the prosocial behavior network while having a sense of meaning and Self-Evaluation function as critical bridges. This study significantly contributes to our understanding of student behavior and mental health. The results suggest that the emergence of prosocial behavior results from interacting with these factors, with having a sense of meaning in life playing a key role in the network. This study provides the first data-driven test to validate how self-acceptance and sense-of-life processes are involved in prosocial behavior. Our findings suggest that individuals with high self-acceptance and sense of meaning may exhibit more prosocial behaviors.

Three indicators were tested in this study. First, calculating the expected effect, emotionality, altruism, and urgency were found to have the highest expected effect. Second, bridge expectancy found that having a sense of meaning and self-evaluation had the highest bridge expectancy impact. Finally, predictability, the multiple squared correlation of each node, was estimated. High predictability indicates that neighboring nodes may provide opportunities for “controllability.” Altruistic behavior had the highest predictability in the model, suggesting that interventions from neighboring nodes, such as anonymity and emotion, may be critical. Therefore, in this study, self-evaluation, having a sense of meaning, altruism, and emotionality were found to be the most important nodes in the network.

The high expected influence of emotionality supports the emotion-driven theory (van Kleef and Lelieveld, 2021), positing that emotional arousal is a key motivator for prosocial behavior. The emotionality node is strongly correlated with altruism (*r* = 0.55) and urgency (*r* = 0.57) in the network, suggesting that emotional empathy may enhance prosocial behavior through two pathways: Firstly, a direct pathway where emotional contagion (e.g., empathic distress at witnessing others’ plight) directly triggers helping behavior (Batson et al., 1997); Secondly, an indirect pathway: Positive emotions (e.g., the joy of assisting) enhance subsequent altruistic motivation through the “broaden-and-build” effect, a psychological process identified by Fredrickson (2001) that broadens an individual’s momentary thought-action repertoire and builds their enduring personal resources.

The centrality of altruism in the network aligns with the evolutionary psychology perspective, wherein altruistic behavior serves as a social bonding agent (Traulsen and Nowak, 2006). Its high predictability (0.66) suggests that interventions targeting anonymity and emotionality may indirectly enhance altruistic behavior through a “ripple effect” (e.g., emotional arousal designs in anonymous donation scenarios).

The high expected influence of urgency not only highlights the role of situational factors but also challenges the traditional “autonomy-normativity” dichotomy. Urgent situations may reshape the decision-making logic of prosocial behavior, a notion that warrants a reconsideration of traditional dichotomies.

The bridge with a sense of meaning exhibited the highest expected influence (0.84), validating the core proposition of existential theory: the sense of meaning in life connects self-perception (e.g., self-evaluation) to behavioral practice (e.g., altruism) through “existential commitment”-a personal dedication to living a meaningful life. From a cognitive integration perspective, individuals with a high sense of meaning are more likely to integrate self-evaluation and altruistic goals into a coherent “life narrative,” (Steger et al., 2008a,b) thereby enhancing the motivational sustainability of prosocial behavior. From a resource spillover perspective, a sense of meaning, as a psychological capital (Hobfoll, 2002), can buffer emotional depletion caused by self-criticism (e.g., “helper fatigue”) and activate cross-situational altruistic tendencies (e.g., proactive intervention in urgent situations).

The bridging role of self-evaluation (0.68) resonates with self-determination theory (Ryan and Deci, 2000). Self-evaluation not only directly relates to self-acceptance (*r* = 0.415) and a sense of meaning in life (*r* = 0.597) but also influences the situational expression of prosocial behavior by moderating the “publicity” node (*r* = 0.262). This underscores the importance of individual differences in shaping prosocial behavior. For instance, individuals with high self-evaluation may be less susceptible to social desirability interference in public helping situations, thereby exhibiting a more stable altruistic tendency.

In this paper, self-evaluation and self-acceptance were the main direct factors of having a sense of meaning in life. The results of this study corroborate with those of previous studies, which have shown that self-acceptance is closely associated with having a sense of meaning in life (Wang et al., 2023). This study further expands the research in this field by exploring the role and origin of self-appraisal in the network of having a sense of meaning in life, based on previous studies. This finding emphasizes the importance of the self-concept in individuals’ lives and how individuals seek unity by integrating different aspects of the self (Reker et al., 2014).

The college years are critical for college students to establish their self-evaluation. At this stage, they face the challenge of independent living for the first time and must form a stable self-concept through self-exploration and identity. In this process, positive self-evaluation can enhance self-esteem and self-efficacy, stimulate their pursuit of goals and intrinsic motivation, and thus enhance the sense of meaning in life. On the contrary, if negative self-evaluation is formed during this period, it may lead to self-doubt and unclear goals, thus weakening the sense of meaning in life (Cao et al., 2023).

The present study found that the sense of having meaning in the sense of the meaning of life influences altruism in prosocial behaviors, based on the theoretical framework of self-determination theory and existentialism, which states that having a sense of meaning satisfies the basic psychological needs for autonomy, relatedness, and competence and that the fulfillment of these needs enhances the intrinsic motivation of individuals to engage in prosocial behaviors (Liu et al., 2013). Existentialism, on the other hand, emphasizes that individuals, in their search for meaning in life, become aware of their connection to others and society. This awareness motivates individuals to transcend their self-interests and demonstrate altruism. In addition, individuals who possess a sense of meaning tend to have a deeper commitment to life, which leads them to feel that their lives are more valuable when they help others, which enhances the tendency to act altruistically (Daniel et al., 1994). The effect of a sense of the meaning of life on prosocial behavior has also been confirmed in previous studies (Chang et al., 2024; Fang et al., 2022; Liu, 2023). The bidirectional association between meaning presence (MLQ-P) and altruism (*r* = 0.37) contrasts with traditional perspectives emphasizing unidirectional predictions from meaning in life to prosocial behavior. For instance, Steger et al. (2008a,b) proposed that individuals with a stronger sense of meaning are more likely to engage in altruistic acts due to existential commitment. Furthermore, our findings align with recent longitudinal studies suggesting reciprocal reinforcement between meaning systems and prosocial engagement. For example, a meta-analysis by Martela and Ryan (2016) on self-determination theory highlighted that fulfilling relatedness needs through prosocial behavior can enhance life meaning, supporting the potential feedback loop observed here. This discrepancy underscores the value of network analysis in capturing dynamic interactions that traditional methods may overlook.

Positive psychology research has shown that positive personal traits, such as optimism, gratitude, and self-acceptance, are positively associated with prosocial behavior (Ma et al., 2022). The centrality of self-acceptance in the network resonates with Neff’s (2011) work on self-compassion, which posits that self-acceptance reduces self-criticism and fosters psychological resources for prosocial actions. While previous studies predominantly focused on linear relationships (e.g., Ryff, 1989), our network model extends these insights by identifying “sense of meaning” as a bridge node. This aligns with Hobfoll’s (2002) conservation of resources theory, where psychological capital (e.g., meaning) facilitates resource spillover from self-perception to altruistic behavior.

This result supports previous research hypotheses that have found that individuals with high levels of self-acceptance are more likely to engage in prosocial behaviors. Therefore, self-acceptance, a lasting and positive social effect that has received increasing attention from researchers, can help individuals establish a positive self-concept, improve self-esteem and self-confidence, and promote psychological health and well-being (Cordaro et al., 2024; Zhang S. et al., 2022; Zhang X. et al., 2022; Miao et al., 2024). In turn, it promotes prosocial behavior, enhances interpersonal harmony, and jointly promotes individual and social well-being. Therefore, improving individuals’ self-acceptance will significantly impact their prosocial behavior.

The findings of this study partially support and extend two theoretical frameworks: Firstly, self-determination theory (Ryan and Deci, 2000): the network model reveals a closed triangle formed by the presence of meaning in life (MLQ-P), self-evaluation (SE), and altruism (Altru.) (MLQ-P↔SE↔Altru.), corroborating the synergistic satisfaction mechanism of basic psychological needs. Secondly, meaning maintenance theory (Heine et al., 2006): the bridging role of possessing meaning (MLQ-P) indicates that the meaning system not only buffers existential anxiety but also promotes the cross-situational generalization of altruistic behavior by endowing it with “ultimate meaning.” This study also identified a bidirectional relationship between meaning in life and prosocial behavior: While longitudinal studies predominantly support a unidirectional prediction from meaning in life to prosocial behavior (Klein, 2017), this study found a strong reciprocal association between possessing meaning in life and altruism (*r* = 0.367), suggesting a potential feedback loop of “meaning-altruistic behavior-meaning reinforcement.” This mechanism can be validated through dynamic network models (e.g., temporal network analysis) in future research.

## 5 Educational recommendations

Based on the results of this study, it was found that fostering positive self-evaluations is essential for promoting altruistic behaviors and building positive social environments during this stage of self-discovery and identity formation in college. These suggest that intervention programs can be used to increase prosocial behaviors among college students. For example, developing college students’ self-evaluation, sense of meaning in life, and altruism in prosocial behavior.

Based on the network analysis findings, this study proposes the following innovative intervention strategies: prioritize core node intervention: design mindfulness-based empathy training (e.g., “Emotion Diary-Helping Action” linked tasks) targeting emotionality and altruism, leveraging their high centrality for network cascade effects; enhance the bridging function of MLQ-P through meaning-oriented life education (e.g., “Life Meaning Workshop”) to promote cognitive integration of self-evaluation and altruistic behavior. Precisely target bridge node intervention: For individuals with low self-evaluation employ cognitive reappraisal techniques (e.g., “Strengths Identification Exercise”) to disrupt the adverse pathway of “low self-evaluation → low sense of meaning in life → low altruistic behavior”; embed meaning cues in emergency simulations (e.g., “Meaning Narrative of Emergency Rescue”), utilizing the bridging role of MLQ-P to enhance the behavioral conversion rate of urgency nodes.

### 5.1 Research shortcomings

This study is the first to integrate network analysis within the framework of positive psychology to systematically investigate the synergistic mechanisms among prosocial behavior, sense of life meaning, and self-acceptance in college students. Theoretical innovations of this work, compared to existing research, are twofold: first, it transcends the traditional variable-centered paradigm to reveal non-linear interaction patterns among positive psychological elements; second, it identifies core facilitators within the network system, providing a basis for developing interventions based on the synergy of psychological resources. The findings are expected to deepen the understanding of the dynamic mechanisms of prosocial behavior and offer theoretical support for positive educational practices that promote individual-societal synergistic development. While this study contributes novel insights to the emerging literature on prosocial behavior, several limitations warrant consideration. First, the reliance on self-report measures introduces potential biases, including social desirability effects and retrospective recall inaccuracies. Although we employed validated scales and statistical controls for standard method variance (e.g., Harman’s single-factor test), self-reports inherently limit our ability to disentangle subjective perceptions from objective behavioral or neurocognitive correlates. Future research could triangulate findings with multimodal data, such as ecological momentary assessment (EMA) or implicit behavioral tasks, to mitigate these biases.

Second, the use of a convenience sampling strategy, while pragmatic for exploratory purposes, may constrain the generalizability of results. Our sample predominantly comprised China’s college students, potentially underrepresenting individuals from diverse socioeconomic, cultural, or clinical backgrounds. This raises concerns about ecological validity. Replication with stratified sampling frameworks or cross-cultural cohorts is critical to verify the robustness of our conclusions. Third, the estimation of the GGMs relied on cross-sectional data, thus precluding any strong inference about potential causal relationships between facets of prosocial behavior.

In summary, although this study has made innovative findings in the field of prosocial behavior, there is still a need for optimization in terms of measurement methods, sample representativeness, and research design to further validate the robustness of the conclusions and expand their generalizability. Future research should adopt multimodal data, stratified sampling, and longitudinal designs. The necessity and potential benefits of longitudinal studies are clear, as they could profoundly investigate the complex mechanisms of prosocial behavior and its causal pathways.

## Data Availability

The original contributions presented in the study are included in the article/supplementary material, further inquiries can be directed to the corresponding authors.
